# Considerations for the Development of Mobile Phone Apps to Support Diabetes Self-Management: Systematic Review

**DOI:** 10.2196/10115

**Published:** 2018-06-21

**Authors:** Mary D Adu, Usman H Malabu, Emily J Callander, Aduli EO Malau-Aduli, Bunmi S Malau-Aduli

**Affiliations:** ^1^ College of Medicine and Dentistry James Cook University Townsville Australia; ^2^ Australian Institute of Tropical Health and Medicine James Cook University Townsville Australia; ^3^ College of Public Health, Medical and Veterinary Sciences James Cook University Townsville Australia

**Keywords:** mobile phone apps, diabetes melitus, self-management, developmental consideration, systematic review

## Abstract

**Background:**

There is increased research interest in the use of mobile phone apps to support diabetes management. However, there are divergent views on what constitute the minimum standards for inclusion in the development of mobile phone apps. Mobile phone apps require an evidence-based approach to development which will consequently impact on their effectiveness. Therefore, comprehensive information on developmental considerations could help designers and researchers to develop innovative and effective patient-centered self-management mobile phone apps for diabetes patients.

**Objective:**

This systematic review examined the developmental considerations adopted in trials that engaged mobile phone applications for diabetes self-management.

**Methods:**

A comprehensive search strategy was implemented across 5 electronic databases; Medline, Scopus, Social Science Citation Index, the Cochrane Central Register of Controlled Trials and Cumulative Index of Nursing and Allied Health Literature (CINALHL) and supplemented by reference list from identified studies. Study quality was evaluated using the Joanna Briggs Critical appraisal checklist for trials. Information on developmental factors (health behavioral theory, functionality, pilot testing, user and clinical expert involvements, data privacy and app security) were assessed across experimental studies using a template developed for the review.

**Results:**

A total of 11 studies (10 randomized controlled trials and 1 quasi-experimental trial) that fitted the inclusion criteria were identified. All the included studies had the functionality of self-monitoring of blood glucose. However, only some of them included functions for data analytics (7/11, 63.6%), education (6/11, 54.5%) and reminder (6/11, 54.5%). There were 5/11(45.5%) studies with significantly improved glycosylated hemoglobin in the intervention groups where educational functionality was present in the apps used in the 5 trials. Only 1 (1/11, 9.1%) study considered health behavioral theory and user involvement, while 2 (2/11, 18.1%) other studies reported the involvement of clinical experts in the development of their apps. There were 4 (4/11, 36.4%) studies which referred to data security and privacy considerations during their app development while 7 (7/12, 63.6%) studies provided information on pilot testing of apps before use in the full trial. Overall, none of the studies provided information on all developmental factors assessed in the review.

**Conclusions:**

There is a lack of elaborate and detailed information in the literature regarding the factors considered in the development of apps used as interventions for diabetes self-management. Documentation and inclusion of such vital information will foster a transparent and shared decision-making process that will ultimately lead to the development of practical and user-friendly self-management apps that can enhance the quality of life for diabetes patients.

## Introduction

### Background

Mobile apps refer to software installed on smart mobile devices that support medical and public health practices [1]. These apps can deliver health care anywhere, subduing geographical and organizational barriers as well as time constraints [2,3]. Their intended use is for diagnosis, self-management, mitigation, treatment or prevention of diseases such as diabetes [4]. Self-management of blood glucose minimizes the risk and health complications associated with the insidious and chronic nature of diabetes [5,6]. Diabetes self-management includes monitoring of glucose level, lifestyle modifications, medication management, prevention of complications and psychosocial care [7]. As a standard, diabetes self-management education is usually provided during outpatient visits; but it has been advocated that most patients require ongoing support to encourage and sustain behavior at the level that can maintain good health [8,9]. Hence, the necessity for a regularly accessible form of diabetes self-management education and support; which can be achieved with the use of mobile apps.

Although, mobile apps are a field that has continually attracted the interest of researchers and has excellent prospects, both for the improvement of health care and economic interest [10,11], comprehensive information on its developmental considerations seem somewhat limited. Studies have reported gaps in the understanding of formal standards and evidence-based approaches employed in the development and evaluation of the effectiveness of mobile apps [12,13].

### Considerations in Mobile Phone App Development

Presently, knowledge about the standard recommended practice for mobile app development for chronic disease management seems divergent and inconclusive. Some studies have reported the benefits of developing mobile apps based on health behavior and communication change theories [14,15]. The main reason for using these theories is to adopt techniques and strategies and help patients embrace healthier lifestyles. Existing models and theories include transtheoretical model [16], social cognitive theory [17], self-determination theory [18], social ecological theory [17] and motivational interviewing [19]. These theories have served as guards in designing mobile app interventions to individuals’ baseline characteristics.

Some authors are of the opinion that the development of health care tools for patient groups such as those with diabetes requires an understanding of current challenges and barriers to self-care [20]. This approach serves as an avenue for exploring users’ needs at a specific time and envisaging what may evolve with time. This can help in visualizing the use of the app as users’ demands change [21,22].

Chomutare et al [23] emphasized in their systematic review that good practice in designing mobile apps requires that inclusion of functionalities be anchored on evidence-based recommendations for the target groups. Furthermore, pilot testing with a target audience and incorporating feedbacks will aid identification of barriers to the usage of mobile apps and enhance the evaluation of its reliability, accuracy, usability, acceptability, and patient adherence [3]. Ensuring the incorporation of evidence-based recommendations and pilot testing into app development for diabetes care will allow for accurate interfaces, interpretations, and evaluation of the effectiveness of the mobile app.

Data privacy and security whereby the users’ information is securely managed is another major developmental consideration [3,24]. Emphasising the use of ‘privacy by design’ approach such as encryption and protocols for anonymous communication and authentication helps to deter unauthorized users from gaining access to patients’ medical data [25,26]. Furthermore, it has been recommended that involvement of clinical experts and multidisciplinary health teams should be an integral part of the developmental and testing process of diabetes mobile apps to ensure that medical guidelines and clinical best practices are followed in the management of diabetes [27].

The various views described above can be labeled as shared decision-making approach to the development of mobile app. Diabetes care and support using this approach in which patients, health care providers, and app developers make health care decision together; taking into account specific evidence as well as specific needs and preferences of patients, has been recommended by various studies because it is seen to produce effective health outcomes [28-30]. Such an approach focuses on patient empowerment, ensuring a transition from a state where patients are only seen as the recipients of care to a position where they also have their opinion considered, and they are allowed to make choices, thereby actively contributing to the decision-making process. Given that the organizational structure within the health care sector now recognizes the patients’ greater role in their health care, this trend should also result in a shift in the process involved in the development of mobile apps. Patient engagement strategies in app development may not necessarily refer to their involvement in the algorithm design but rather in the incorporation of procedures that meet patients’ expectations through the consideration of their experiences, needs, reasons for engagement and satisfaction with the usage of the app.

Mobile apps have been proven to be a useful lifestyle modification tool for providing ongoing individual self-care support for diabetes management and facilitating regular monitoring for improved health outcomes [31-36]. However, previous reviews have focused mainly on assessing the effectiveness of mobile apps to support diabetes self-management [11,33,34,36,37]. A mixture of shared decision-making approaches that include developmental considerations such as health behavioral theories, user and clinical expert involvement, pilot testing and data security are essential to help solve the problems of poor engagement experience and ineffective use of mobile apps [38].

The inclusion of robust, reliable and repeatable system design that involves end users early in the developmental consideration process will enhance ongoing support which is crucial to sustaining progress made by diabetes patients in their self-management [39]. To the best of our knowledge, no other study has collated evidence on the factors taken into consideration in the development of such apps. This evidence will further aid the advancement of evidence-based development and evaluation of mobile apps for effective diabetes management.

This systematic review aims to evaluate the factors taken into consideration in the development of mobile phone-based apps used as self-management interventions in experimental trials of adults with diabetes. Also, the review compares these mobile app developmental factors with their impact on the key clinical outcome variable glycosylated hemoglobin (HbA_1c_). For this study, the developmental factors considered are categorized into the following: (1) Health behavioural change theory, (2) Function/Functionality (comprising documentation, analytics, reminder, and education), (3) Users involvement, (4) Clinical expert involvement, (5) Data security and privacy consideration, and (6) Pilot testing. These factors were considered based on extensive literature search and ingeminate brainstorming sessions among co-authors, with a focus to provide a guide on factors to consider in the development process of mobile app for diabetes self-management precluding the use of such apps in a full trial.

## Methods

This systematic review was conducted following the Preferred Reporting Items for Systematic Review and Meta-Analyses (PRISMA) statement [40]. Assessed developmental considerations are based solely on author reported descriptions directly available in the selected studies or referenced in another published article. For this review, we defined mobile phone apps as apps that are downloadable to mobile phones and take data inputs from users with a focus on improving one or more aspects of diabetes self-management domains.

### Data Sources and Search Strategy

Published literature sources were identified by searching Medline, Cumulative Index to Nursing and Allied Health Literature (CINAHL, EBSCOhost), Scopus, Social Science Citation Index and Cochrane Register of Controlled Trials (CENTRAL) databases. In order for search results to have the maximum possible coverage, the combination of the following terms and medical subject headings were used during the search: (‘‘Type 1 diabetes mellitus’’ OR ‘‘Type 2 diabetes mellitus’’ OR diabet* OR IDDM OR NIDDM) AND (‘‘Mobile applications’’ OR , Smartphone* OR ‘‘app’’ OR ‘‘cellular phone’’ OR ‘‘mobile app’’ OR ‘‘portable electronic applications’’ OR ‘‘portable software application’’ OR ‘‘text messages’’). Searches were done between 5th-29^th^September 2017. Searches were supplemented by manual searching of reference lists of identified studies.

### Selection Criteria

Selected studies were any randomized controlled trial (RCT), quasi-experimental study, or pre-post study evaluating the use of mobile apps for self-management in patients (≥ 18 years) with type 1 or 2 diabetes. Studies included were those that used mobile phone-based app intervention which allows real-time interaction between patients and the software. Such interactions include input from the user (which may or may not allow for reinforcement of personalized or general advice), goal setting, data analytics, decision support or reminders to improve diabetes self-management. Strict inclusion criteria were applied to streamline and capture only diabetes interventional studies. Therefore, to ensure review of fully functional apps used as an intervention for diabetes management, only trials that evaluated at least one glycemia index of glycosylated hemoglobin (HbA_1c_) or blood glucose levels as primary outcome were included. Selected studies were those published in the English language but not restricted to patients of any particular race.

Exclusion criteria included: (1) technological interventions not including mobile phone based app, for example systems which require patients to input data into a Web-based server for review by clinician or researcher, (2) systematic reviews, meta-analyses, conference papers or letters, (3) pre-diabetes, gestational and secondary diabetes, (4) obesity, (5) software solutions mainly for insulin pumps only, (6) studies on mixed populations of adults and children, and (7) studies still ongoing that presented interim results only.

### Data Extraction

The titles and abstracts of all identified references were reviewed by the first author (MD). References that did not meet all of the inclusion criteria were excluded. The full-text article of all relevant references was retrieved and assessed. Data were extracted from each selected studies using an electronic form purposely developed for this review. All authors checked the extracted data for consistency. Discrepancies were resolved through discussion.

### Quality Assessment

Assessment of study quality was performed by one author (MD) in consultation with a second author (BMA). The quality was evaluated using Joanna Briggs Institute’s pre-designed standardized critical appraisal tools [41]. For the RCTs the following criteria were considered: (1) true randomization of assignments, (2) allocation concealment, (3) blinding of outcome assessors, (4) intention-to-treat analysis, and (5) appropriateness of trial design. Criteria considered for the quasi-experimental trial included (1) clear description of cause and effect, (2) presence of a control group, and (3) pre and post intervention outcome measurements were assessed. For all studies, criteria included (1) details of similarity in baseline characteristics, (2) identical treatment for groups with the exception of intervention of interest, (3) degree and description of follow up, (4) similarities in group outcome measurements, (5) reliability of outcome (primary outcome of HbA_1c_ or blood glucose levels), and (6) suitability of statistical analysis were evaluated. Blinding of participants and personnel were part of the quality criteria in the tools but were omitted and termed non-applicable since the nature of the intervention under study makes it difficult to achieve blinding. All criteria on the tools were scored on a 2 point scale: Yes (1 point) or no or unclear (0 points). When adding all quality criteria, the maximum obtainable scores was 11 for the RCTs and 9 for the quasi-controlled trials. Depending on the number of criteria met by each study, the quality of each study was graded as High (≥7 points), moderate (4-6 points) or low (≤3 points). Disagreement were resolved through discussion among authors.

## Results

### Selection of Studies

**Figure 1:**
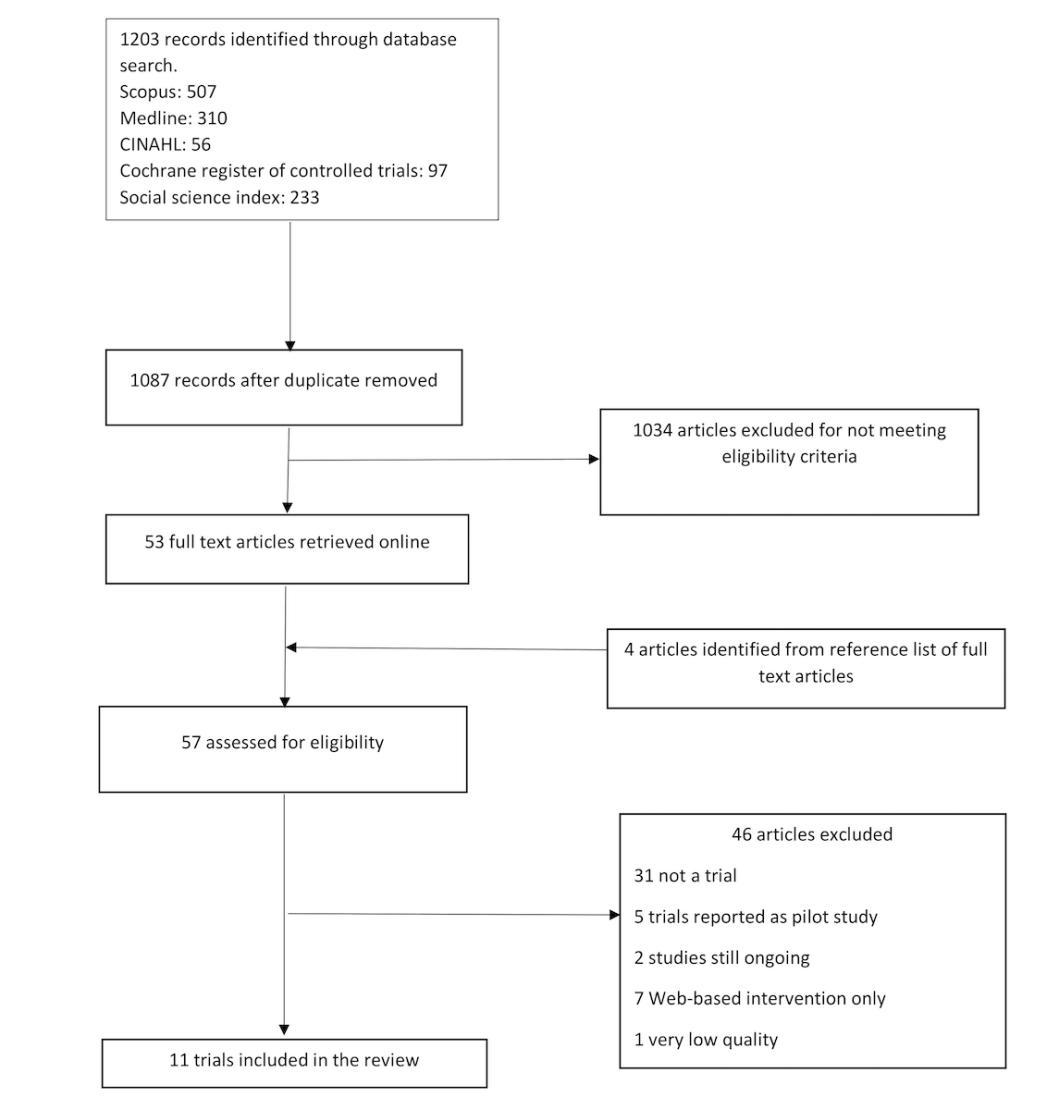
Flow diagram of the study selection process.

The initial search from the 5 databases identified 1203 articles which included 116 duplicates that were removed. Based on the review of the titles and abstracts, 53 articles were potentially relevant. The full text of these articles was retrieved for further examination, and their references were manually screened to identify articles that were not included in the original search. This process yielded 4 additional articles. After reading the full articles, 12 studies met the set inclusion criteria. The studies by Quinn et al [42,43] reported on the same study population, with different group classifications. The studies by Rossi et al [44,45] engaged the same app but in different study populations. Therefore, 11 RCTs and 1 quasi-experimental study were eventually included. An adapted PRISMA (Preferred Reporting Items for Systematic Reviews and Meta-Analyses) flow-chart of study selection is shown in Figure 1.

### Methodological Quality Assessment

There were 7/12 (58.3%) true randomization trials [44-50], and 2/12 (16.7%)trials had unclear evidence for their randomization method as there was insufficient detail to make a judgment [42,43]. Allocation concealment was documented in only 1/12 (8.3%) study [48].

A total of 7/12 (58.3%) studies reported an intention-to-treat analysis of their data [43-47,50,51]. There was 1/12 (8.3%) study that reported the use of a linear mixed methodology which allowed the inclusion of all randomized participants [48]. A total of 9/12 (75.0%) studies had details of attrition with reasons for drop out balanced across groups [42-46,48-50,52].

All studies had similar and reliable HbA_1c_ measure. All studies, except 1/12 (8.3%) by Istepanian et al [47], were judged to be appropriate in their statistical analyses and trial designs. Overall, 10/12 (83.3%) studies were graded as high quality because they met 7-9 criteria of the grading tool, 1/12 (8.3%) study met 6 of the criteria and was graded as moderate [47], and the last study (1/12, 8.3%) met only 2 quality criteria [53], was graded as poor and removed from the review.

### Characteristics of Included Studies

The 11 studies selected evaluated 9 mobile apps and were published between 2009 and 2016. A total of 10/11 (91.1%) studies were RCTs, while 1/11 (9.1%) was a quasi-experimental study [52]. Participant numbers ranged from 54 [50] to 213 [43]. There were 4/11 (36.4%) studies which focused on type 1 diabetes [44-46,48], 6/11 (54.5%) studies were specific to type 2 diabetes while 1/11 (9.1%) study [47] involved both type 1 and 2 diabetes patients. Intervention duration for 8/11 (72.7%) studies ranged from 2 to 10 months, while the remaining 3/11 (27.3%) studies [42,43,51] had their follow up period extended to 1 year. Study locations were from four geographic regions including Europe (6/11, 54.5%), Oceania (1/11, 9.1%), Asia (2/11, 18.2%) and America (2/11, 18.2%).

All studies had major interventions using a mobile app. A total of 2/11 (18.2%) studies had 2 intervention groups [46,51] and another 2 studies had 3 intervention groups [42,43].

HbA_1c_ was the primary outcome measure in all trials. A total of 5/11 (45.4%) studies reported a positive and statistically significant improvement in HbA_1c_ in the intervention group [43,46,48-50]. A total of 5/11 (45.4%) studies had HbA_1c_ reduction in both the intervention and control groups [42,44,45,51,52]. While in 1/11 (9.1%) study, HbA_1c_ remained unchanged between the intervention and control groups [47]. A summary of these characteristics is shown in Multimedia Appendix 1.

Multimedia Appendix 2 and Multimedia Appendix 3 detail the developmental factors considered in each of the reviewed studies and the resulting key clinical outcome (HbA_1c_).

### Health Behavioral Theories

Only 1/11 (9.1%) study [49] reported on health behavioral theories. Specifically, motivation behavioural skills model was used for the formulation of an automated personalised feedback message content of the mobile app.

### Functions of Mobile Apps

It was apparent from the review that functions of the mobile apps were diverse. However, documentation for self-monitoring of blood glucose (BG) either manually or through wireless transmission from BG meter was present in all studies. A total of 8/11 (72.2%) studies had mobile apps with capacity for diet management [42-46,48,50,51]. Three studies incorporated blood pressure function in their mobile apps [49,50,52]. There were 7/11 (63.6%) studies which had a physical activity function [44-46,48-51] and 2/11 (18.2%) studies incorporated weight tracking function [49,50]. There were specific functions to log or calculate insulin dosages in mobile apps employed in the 4/11 (36.3%) studies with type 1 diabetes participants [44-46,48]. A total of 2/11 (18.2%) studies reported a general medication log function in their mobile apps [42,43].

With the exception of 4/11 (36.4%) studies, all others (7/11, 63.6%) had capacity for mobile apps to allow patients to analyse logged data. These 4 studies had their logged data transferred to a web/cloud storage and analysed by either the researcher or the health provider [42,43,47,52].

There were 6/11 (54.5%) studies that utilised mobile apps with an educational function. Half 3/6 (50.0%) of the studies provided education as a personalised real-time automated educational feedback specific to logged data [42,43,49], while the other 3 provided a general information page [44,45,51].

A total of 6/11 (54.5%) studies utilized a mobile app with a reminder function [44,45,47,48,50,51].

### Users’ Involvement

There was only 1/11 (9.1%) study [51] that clearly described users’ involvement in the design of its mobile app. It reported an iterative design process involving 12-15 diabetes patients using the approach of focus group meetings, semi-structured interviews, usability testing, questionnaires and paper prototyping. This approach generated the design requirements and answers to research questions [20].

### Clinical Expert Involvement

There were 2/11 (18.2%) studies [42,43] which used the same mobile app and engaged the opinions of clinical experts in the field of diabetes during its development and design. The studies reported that the mobile app development involved an Endocrinologist and a Credentialed Diabetes Educator [54].

### Data Security and Privacy Consideration

Report on data security and privacy varied among the studies with limited elucidation of information in most cases. In 2/11 (18.2%) studies [42,43] the authors reported a real time capturing of self-monitored blood glucose data into a Health Insurance Portability and Accountability Act-compliant secured Web-based system [54]. In 1/11 (9.1%) study, measured data from participants were transmitted to a server. With each new measurement the patient profile was updated allowing controlled access to patients’ data and record history [50]. Transfer of mobile app data into a secured central server was the only information provided by Charpentier et al [46].

### Pilot Testing of Mobile Apps

A total of 7/11 (63.6%) studies provided information with regards to pilot testing. Of these, 2/11 (18.2%) [42,43] reported three months test running of the mobile app on 30 patients with type 2 diabetes with the aim of evaluating the impact on HbA_1c_ and satisfaction of patients with the technology [54]. Likewise, 1/11 (9.1%) study [46] reported a 4-month open label observational pilot study on 35 type 1 diabetic patients with the aim of confirming if the use of the mobile app resulted in good control of post prandial blood glucose readings [55]. Only 1/11 (9.1%) study [50] reported a one-month piloting on 11 type 2 diabetes patients to assess usability and impact of the mobile app on HbA_1c_ outcomes and home blood pressure monitoring [56]. In 2/11 (18.1%) studies [44,45], 2 pilot programs were reported through a citation in another article. The first was with the use of a questionnaire to assess the feasibility and acceptability of the mobile app. The second was a 9-months follow up of 41 patients using the mobile app under routine clinical practice condition with the aim of investigating its effectiveness on metabolic control [57]. Lastly, 1/11 (9.1%) study [51] reported a 12 months pilot testing on 12 persons with type 2 diabetes [20].

## Discussion

### Theoretical Basis

Our review shows that most of the studies did not discuss consideration for health behavior theories in their mobile app development. The lack of report on theoretical basis may be as a result of reliance on evidence-based guidelines that relates to the essential self-care activities in people with diabetes to predict good outcomes [58]. While it is necessary for mobile apps to be guided by health behavioral theories, the current theories appear incapable of answering most of the questions likely to arise when mobile apps are employed as health interventions [14]. Dunton and Atienza [59] reported that current health behavior theories have not been able to incorporate within-person differences which allow for intra-individual tailoring of interventions. Boorsboom et al [60] noted that between people theories do not imply, test or support causal factors valid at the individual level. Therefore, there is a need for more research into intra-individual non-static regulatory models which can be incorporated in the development of mobile technology-based health behavioral interventions.

### Functionalities of Mobile Apps

All the 11 trials reviewed in this study included mobile apps with documentation/monitoring component, where self-documentation of blood glucose readings was the most common. Only 3 studies used mobile apps that offer automated direct data transfer of blood glucose values from the glucometer or data from other measuring devices [47,50,51]. This corroborates the report by Demidowich et al [61], where only four of the 42 mobile apps studied offered direct data input from glucometer. Data entry is often perceived as a persistent burden in chronic disease management [39]. Therefore, it is imperative that data entry in mobile apps be as spontaneous as possible, requiring little time and effort to use [62]. Mobile app developers should prospectively consider including an interface between the app and biomarker measuring devices which allow users to automatically log measurements. Such interface may include Bluetooth which enables portable electronic devices to connect and communicate wirelessly [63]. The success of using this interface was demonstrated in the studies by Waki et al [50] and Holmen et al [51].

Data analytics as an app feature was included in only 7/11 (63.3%) studies. A consumer-directed software such as mobile app is better incorporated with functions that enable users to enter, analyze their health parameters and view graph trends and statistics. This can improve the patient’s ability to observe the impact of their lifestyle and behavior on health indicators, access trends and even predict health outcome measures [64]. Additionally, decision-making and problem-solving skills of patients can be improved when mobile apps include visualization techniques such as color-coded charts or graphs which indicate when biomarkers, food carbohydrate component and physical activity are out of recommended range [65]. It is essential that analytic functions be dynamic, easily accessible and able to project trends to predict individual improvement in self-care activities which may invariably lead to better health outcomes [66,67].

Despite the emphasis by published guidelines for the need for ongoing patient education [7], very few studies used mobile apps that have education as a functionality. This finding is corroborated by another review where the authors confirmed personalized education as an underrepresented feature in diabetes mobile apps [23]. Patients may have difficulty consulting with their diabetes educators or other health care professionals, due to lack of time, financial constraints, and other limitations. Hence, an app with an educational component can supplement health care provider diabetes education and reinforce information about the importance of self-management and complication prevention. This can serve as an avenue for continual patient empowerment to successfully deal with the disease. However, it is essential that the personalized educational feedback and advice provided in mobile apps are accurate. This is especially true for those that are automatically generated because monitoring mobile apps pose serious harm to the patients if they fail to function as intended [68].

A total of 6/11 (54.5%) studies reported using mobile apps with reminder function either in the form of prompting to measure missed blood glucose readings or alerts for appointments scheduled for the assessment of complication [44,45,47,48,50,51]. They are sometimes referred to as ‘push technology’; which enables messages to be delivered without any effort on the part of the recipient [69]. Such reminders can be in the form of text message, alarm, email, automated voice call or image message. Other review has illustrated the benefits of an alarm reminding patients to carry out their health activities [70]. Another study revealed improvement in treatment adherence as patients get fascinated using reminders to handle their health care activities [71].

### Users’ Involvement

Similar to an earlier review by El-Gayar et al [72] on the adoption of user-centered designed principles in mobile apps, only one study [51] documented inquiry into users’ expectations and perceived needs in the app developmental phase. Users’ involvement in design process increases the success rate of computerized system usability [73], as it is essential to understand the reasons for use and user requirements [74,75]. In contrast, a design process lacking the involvement of users in the design loop will fail to recognize the particular odds and problems in the use of the intervention [76]. Design processes can use research tools such as questionnaires, focus group discussions, and personal interviews. These help to seek users’ requirements, preferences, understand current challenges and barriers to self-care and subsequently incorporate the findings into the design process. Incorporation of feedback during app design process can help in producing a more user-friendly application and encourage long-term user engagement.

### Clinical Expert Involvement

Many of the apps reported in the studies reviewed were designed without the involvement of health care professionals, and this observation is supported by an earlier review [77]. Involvement of health professionals in diabetes mobile app development can assure the quality of health information and support provided by such apps [78]. This is especially important in mobile apps involving advice on insulin dosing. It has to be mentioned that the 3/11 (27.2%) studies in this review which used mobile apps to assist participants in calculating insulin dosage failed to report whether clinical experts were involved in the development of these apps, even though HbA_1c_ levels in the intervention groups were not significantly lower compared to the control groups [44-46]. This finding highlights possible issues with the effectiveness, efficiency, and relevance of these mobile apps to users’ health security. Insulin overdose in diabetics can result to severe hypoglycemia and coma while under-dose can cause diabetes ketoacidosis; both can have fatal consequences [79,80]. Participation of health professionals in the development of diabetes mobile apps may decrease the likelihood of such fatal occurrences and protect consumers from incorrect and misleading information. Furthermore, clinical expert involvement in diabetes mobile app development will foster avoidance of legal implications surrounding noncompliance to regulatory and medical standards that relate to digital health services especially those which empower people to track, manage and make decisions about their health [81,82].

### Data Security and Privacy

Information on data security and privacy considerations in mobile app development were lacking in many of the trials in this review. Late consideration of privacy and security are app developers’ errors that cannot be underestimated. Medical data breaches resulting from failed security attract huge financial implications (such as costs associated with a pecuniary penalty, potential liability claim, lost brand value, responding to lawsuits, negative press statements and essentially loss of patients’ and health care providers’ trust) for non-compliant organizations [82,83]. Studies have revealed that some users are concerned about the privacy of their personal health information stored on an electronic device [84,85]. Procedures to maintain health data privacy and security to avoid data breaches must, therefore, be considered during mobile app design. Encrypted storage which ensures logged data are protected against malicious attack is a security approach to protecting health data on mobile apps [86]. Furthermore, the privacy of users’ information can be ensured through user authentication or enforcement of password requirements [86], and this can protect users’ health data in case of mobile phone loss.

### Pilot Testing

There were 5/11 (45.5%) studies that failed to report on pilot testing of their apps before use in the trial. A previous study also reported that most health apps do not offer patients ample opportunity for feedback on the level of satisfaction and usability of the product [87]. The importance of pilot testing mobile apps cannot be overemphasized. Apart from serving as an avenue for testing the impact of the app on glycemic control pilot testing can assess its user-friendly capacity and adherence for use as a self-management tool.

### Developmental Factors Considered in Mobile Apps and the Key Clinical Outcome (Glycosylated Hemoglobin)

Multimedia Appendix 2 and Multimedia Appendix 3 show an overall evaluation of the developmental factors considered in the design of the mobile apps used in the reviewed studies and the resulting critical clinical outcome (ie, glycosylated hemoglobin, HbA_1c_). Multimedia Appendix 3 highlighted 5/11 (45.5%) studies that had intervention groups with significantly improved HbA_1c_. A comparison of these 5 studies showed that educational functionality was present in all. For example, 3/5 (60.0%) studies provided the educational information directly through the mobile app [43,49,50] while 2/5 (40%) provided additional text messaging or teleconsultation [46,48]. It is likely that the similar outcomes observed in these studies were partly due to similitude in the provision of self-management education to participants, as digital tools with decision support features such as education have been proven to have the capacity to enhance self-management outcomes [88]. This finding demonstrates the importance of consistent and ongoing provision of self-management education to people with diabetes. Diabetes education and diabetes management are inseparable because every patient would benefit from education in self-management. Therefore, in addition to other essential functionalities in mobile apps that support diabetes care, the inclusion of education functionality will provide the recommended ongoing support to promote the importance of self-management, build patient skills, increase motivation for self-care and ultimately improve glycemic control [89,90].

Furthermore, 3/5 (60%) studies with significant improvement in HbA_1c_ reported on pilot testing of their mobile apps before use in the full trial [43,46,56]. It is possible that excellent efficacy observed in these studies was due to pilot testing. Among other reasons, an essential aim of pilot testing a technology is to establish its usability. Usability testing of a mobile app examines end users’ satisfaction and has been identified as one of the factors that determine its efficacy and success of users’ engagement with it. [91].

### Implication for Practice and Future Research

Much work is needed to address challenges limiting the documentation and the implementation of developmental factors in the design of mobile apps for diabetes management. The use of mobile phone interventions in which the developmental design are not explicitly documented is likely to result in a non-replicable app with significant levels of wasted resources. Therefore, future work is required to promote the development of evidence-based apps research and clinical use. These mobile apps should focus on integrating functions to core diabetes self-management practices and primarily with the provision of self-management education. Additionally, integrating theories of health behavioral change, users, and clinical experts’ involvement while ensuring data privacy and security are essential factors to be considered in the development of future mobile apps.

### Limitations of This Review

There are limitations to be considered when interpreting and extrapolating the findings of this systematic review. The results of this review were dependent on the terms used in the search strategy and the efficiency of the search engines used. An attempt to overcome this limitation was ensured by choosing common terms and combination of terms usually used in the literature review on mobile health apps. This review considered only trials that were reported in the English language with strict inclusion criteria and so the number of articles that met the study criteria was small, and this limits the ability to generalize the findings. Also, the process of extracting the data presented some risk of error and uncertainty because some studies were not explicit about their developmental considerations, and it is easy to miss or misunderstand some development description either reported directly within the article or referenced. However, to avoid this occurrence, the authors ensured that the assessment process involved independent verification and all pitfalls that might invalidate the findings were avoided. Despite these limitations, this review provides valuable information to future researchers and developers of mobile apps for diabetes management on the necessary factors to consider during app development.

### Conclusion

This systematic review has presented the crucial steps that need to be taken in mobile app development to support effective self-management for people with diabetes. Most of the studies in this review offer a limited and non-expository degree of information on the factors considered in the development of the apps employed.

The main stakeholder in diabetes management is the patient. Shared decision-making between diabetes patients, health care professionals, and app developers can result in improved management. Therefore, this should be the basis for the development of mobile apps for diabetes support. Shared decision-making can be achieved through the process of patient and clinical expert involvement, ensuring data security and privacy, pilot testing and integration of core functions that support all aspects of diabetes self-care activities as indicated by evidence-based guidelines. Continual integration of these processes during app development (before actual use in clinical trials) will ensure that specific needs of diabetic patients are met in the finally developed app, and this will ultimately improve diabetes support, self-management and clinical outcomes for the patients.

## Appendix

Multimedia Appendix 1 Study Characteristics.

Multimedia Appendix 2 Some of the developmental considerations in the reviewed studies.

Multimedia Appendix 3 Other developmental considerations in the reviewed studies and key clinical outcome-glycosylated hemoglobin.

## Abbreviations

BG: blood glucose
CINAHL: Cumulative Index to Nursing and Allied Health Literature
HbA_1c_: glycosylated hemoglobin
HIPAA: Health Insurance Portability and Accountability Act
IDDM: Insulin Dependent Diabetes Mellitus
Medline: Medical Literature Analysis and Retrieval System Online
NIDDM: Non-Insulin Dependent Diabetes Mellitus
PRISMA: Preferred Reporting Items for Systematic Reviews and Meta-Analysis
RCT: Randomized Controlled Trial
