# Supplementary material for: Considerations for the Development of Mobile Phone Apps to Support Diabetes Self-Management: Systematic Review
Source: JMIR Mhealth Uhealth. 2018 Jun 21;6(6):e10115. doi: 10.2196/10115 (PMC6035345; doi:10.2196/10115)
Supplement: Multimedia Appendix 1 [file mhealth_v6i6e10115_app1.pdf]

Multimedia Appendix 1: Study characteristics

Author (s), Year	Study Design, Location, Type of diabetes (DM[g]), Total number of participants (N[h]), Duration (in months), Attrition rate	Stated aim	Intervention/Comparator	Summary of $HbA1_c$[c] result (Mean, SD[i])	Quality (high[a], medium[b])
**Kirwan, 2013 [48]**	RCT; *Country:* Australia; *Type of DM:* Type 1; *N*=72; *Duration:* 6 months; Attrition: 19 (26.3%)	To examine the effectiveness of a freely available smartphone app combined with text message feedback from a certified diabetes educator to improve glycemic control and other diabetes related outcomes in adults with type 1 diabetes	1 intervention group who received: (i) a freely available mobile app that allow users to manually enter BG[d] levels, insulin dosages, other medications, diet and physical activities (ii) one personalized SMS[j] per week. ***Comparator***: Usual care	Significant $HbA1_c$ improvement in the intervention group [from mean 9.8% (SD 1.18) to mean 7.8% (SD 0.75)] compared to the control [from mean 8.47% (SD 0.86) to mean 8.55% (SD 1.16)] after 9 month follow up (*P*<.001).	1
**Quinn, 2011 [43]**	Cluster RCT; *Country:* USA; *Type of DM*: Type 2; *N*= 213; *Duration:* 12 months; Attrition: 50 (23.4%)	To test whether adding mobile app coaching and patient/provider web portals to community primary care compared with standard diabetes management would reduce $HbA1_c$ in patients with type 2 diabetes	3 intervention groups: (A). Who all received: (i) a mobile phone, app, BG[d] meter, one year unlimited mobile phone data / service plan, and web based portal. App allowed participants to enter BG[d], CHO[f] consumed and medications. (ii) In response to logged data, participants received automated real time educational, behavioural and motivational messages. (iii) Received text messages on portal as	1.9 % and 0.7% mean $HbA1_c$ decline in the maximal intervention and control groups respectively. Significant $HbA1_c$ improvement was observed between the maximal intervention and control group only (*P*=.001). No significant changes	1

			a supplement to automated messages. (iv) Feedbacks summaries of entered data and self-management action plan every 2.5 months which also serves as pre-visit summary for patient's next visit to HCPs[i].<br>B. In addition to the above; group 2 HCPs[i] have access to a portal if they choose to review patients unanalysed data.<br>C. Group 3 HCPs[i] have access to review patients' analysed data on the web portal. ***Comparator***: Usual care	between other groups	
**Charpentier, 2011 [46]**	Parallel group RCT; Country: France; Type of DM: Type 1; *N* = 180; Duration: 6 months; Attrition: 7 (3.9%)	To evaluate the efficacy of mobile app in improving metabolic control in poorly controlled type 1 diabetes patients	2 intervention groups.<br>(A). Both groups were provided with a smartphone and mobile app which can provide (a). Bolus insulin dose based on logged, pre-meal BG[d], CHO[f] and anticipated physical activity. (b). Plasma glucose targets. (c). Algorithm for the adjustment of CHO[f] ratio and basal insulin doses.<br>(B) Group 1 had a quarterly follow up hospital visit.<br>(C). Group 2 received teleconsultation every 2 weeks and a follow up visit after 6 month.<br>***Comparator***: usual care plus use of paper log book and required to attend two follow up hospital visits	The intervention group 1 (app only) had 0.67% improvement in mean $HbA1_c$ over the control group (*P*=.022) while the intervention group 2 (app + teleconsultation) had a significant 0.91% improvement in mean $HbA1_c$ over the control group. (*P*=.002)	1
**Quinn, 2016 [42]**	Cluster group RCT; *Country:* USA; *Type of DM:* Type 2; *N* = 118; *Duration*: 12 months;	To determine if there were differences in the impact of mobile intervention in younger adults (<55 years old) versus older adults (≥55 years) within those	1 intervention group (with older and younger subgroup) who received: (i) a mobile app and HCP[i] decision support. App allowed participants to log BG[d], CHO[f] and medications. (ii) Automated real time education, behavioural and	Decreased mean $HbA1_c$ in both groups. The intervention arms [older and younger patients] had $HbA1_c$ decline of 1.8% and 0.2%	1

	Attrition: Not reported; this was a subset analysis of Quinn 2011	below 64 year old. To understand if there were difference for the cohort nearing 65 years old.	motivational messages in response to logged data. (iii) Logged data were intermittently reviewed by a diabetes educator with whom patients were allowed to have telephone communication. (iv) Web portal to receive supplemental messages, access personal health records and also communicate with HCP[i]. (v) A mobile phone, 1 year unlimited data /service plan and the study app. (vi) Received electronic action plan for diabetes management every 2.5 months and pre-visit summary for physician office visit. ***Comparator*:** Usual care	respectively while the control group [older and younger patients had HbA1$_c$ decline of 0.3% and 0.1% respectively] (*P*=.0001)	
**Waki, 2014 [50]**	Parallel group RCT; *Country:* Tokyo; *Type of DM:* Type 2; *N* = 54; *Duration*: 3 months; Attrition: 5 (9.3%)	To access the usability of a remote health monitoring system and especially its impact on modifying patients lifestyle and clinical outcomes	1 intervention group who received: (i) A smart phone with app. (ii) Glucometer, Bluetooth-enabled BP[e] monitor, pedometer and scale. All devices were paired with a unique communicator that transmit the measured data through wireless network to a mobile app server. ***Comparator***: Usual care	Intervention group had significant improvement (0.4%) in HbA1$_c$ [from mean 7.1% (SD 1.0) to mean 6.7(SD 0.7%)] compared to 0.1% increase in HbA1$_c$ in the control group [mean 7.0% (SD 0.9) to mean 7.19% (SD 1.1)] (*P*=.019).	1

**Orsama, 2013 [49]**	Parallel group RCT; *Country:* Finland; *Type of DM:* Type 2; *N* = 56; *Duration*: 10 months; *Attrition:* 8 (14.3%)	This research involved the development and evaluation of a mobile telephone based remote patient reporting and automated telephone feedback system, guided by health behaviour change theory, aimed at improving self-management and health status in individuals with type 2 DM.	1 intervention group who received: (i) a mobile app for reporting BP[e], weight and physical activities. In specific cases, BG[d] meter was provided to those with high $HbA1_c$ values. (ii) Measuring devices for BP[e], weight and physical activity (pedometer). (iii) Automatically generated health promotion information, motivation and behavioural skills feedback messages linked to patient remote self-reported health parameters. (iv) Participants had access to personal health records such as medication, laboratory data and personal care plan. ***Comparator***: Usual care	A significant 0.4% improvement in mean $HbA1_c$ in the intervention group [from -0.67% to -0.14%] compared to 0.036% in the control group [from -0.23% to 0.30%] (*P*=.03).	1
**Kim, 2014 [52]**	Quasi experiment ; *Country: Korea*; *Type of DM:* Type 1; *N* = 73; *Duration*: 3 months; *Attrition:* 3 (4.1%)	To assess the efficacy of the smartphone-based health app for glucose control and patient satisfaction with the mobile network system used for glucose self-monitoring	1 intervention group who received: (i) a mobile app to log BG[d] data which were automatically sent to the medical team for analysis and to provide recommendations at least once a week. (ii) In the events of hypoglycaemia in participants or failure to record BG[d] measurements, the medical team called such participant to change insulin dose or recommended an early visit to the hospital. ***Comparator:*** usual care	Non-significant improvement in $HbA1_c$ in the intervention group [from mean 7.7 % (SD 0.7) to mean 7.5% (SD 0.7) (*P*=.077) and the control groups [from mean 7.7% (SD 0.5) to mean 7.7%(SD 0.7)] (*P*=.093)	1

**Rossi, 2010 [44]**	Parallel group RCT; *Country: Italy, England and Spain*; *Type of DM:* Type 1; *N*= 130; *Duration*: 6 months; *Attrition*: 11 (8.5%)	To evaluate whether the use of a mobile software intervention could be effective in improving metabolic control in type 1, while avoiding weight gain and reducing time devoted to education. Also, to investigate the extent to which the mobile software could affect the quality of life	1 intervention group who received: (i) a mobile app to log data on BG[d] and insulin dose. (ii) With logged information on physical activity, glycemic target and specific events, the app can calculate and suggest appropriate insulin dose and daily carbohydrate intake. ***Comparator***: usual care	Similar 0.5% reduction of $HbA1_c$ in both groups [Intervention: from mean 8.2% (SD 0.8) to mean 7.8% (0.8) and control: from mean 8.4% (SD 0.7) to mean 7.9% (SD 1.1)] (*P*=.68)	1
**Holmen, 2014 [51]**	3 arms prospective RCT; *Country: Norway*; *Type of DM:* Type 2; *N* = 151; *Duration*: 12 months; *Attrition:* 31 (21%)	To test whether the use of mobile phone based self-management system used for 1 year with or without health counselling by a diabetes specialist nurse for the first 4 months could improve $HbA1_c$, self-management and health-related quality of life with usual care	2 intervention groups.<br>A). One group received a mobile phone app only<br>B). The other received an app plus health counselling via telephone by a diabetes nurse.<br>***Comparator:*** usual care	$HbA1_c$ decreased in all three groups but no difference between groups. No specific data reported	1
**Rossi, 2013 [45]**	Parallel group RCT; *Country: Italy*; *Type of DM:* Type 1; *N*=127; *Duration*: 6 months; *Attrition:* 15 (11.8%)	To compare the efficacy of a diabetes interactive diary (DID) versus usual care on metabolic control, hypoglycaemia and quality of life	1 intervention group who received: (i) a mobile app and attended a course on usage. App was used to estimate CHO[f] content in meals and prandial insulin doses were adjusted based on app algorithm. (ii) Education on hypoglycaemia<br>iii). BG[d] meter.<br>***Comparator***: usual care and received a BG[d] meter	Non-statistical $HbA1_c$ reduction in both groups. Intervention [decrease of mean 0.41% (SD 0.11)] and control [decrease of mean -0.48% (SD 0.11)] (*P*=.73)	1

**Istepanian, 2009 [47]**	Parallel group RCT; *Country: London*; *Type of DM:* Type 1&2; *N* = 137; *Duration*: 9 months; Attrition: 50 (36.5%)	To evaluate m-health system against usual care	1 intervention group who received: (i) a mobile phone app (ii) 2 hours education on general diabetes care and self-BG[d] monitoring (iii) BG meter. ***Comparator***: usual care and received 2 hours education on general diabetes care and self-BG[d] monitoring	There were no differences in mean $HbA1_c$ between the intervention and control groups:7.9% and 8.2% respectively (*P*=.17)	2

[a]1: high.
[b]2: moderate.
[c]$HbA1_c$: glycosylated hemoglobin.
[d]BG: blood glucose.
[e]BP: blood pressure
[f]CHO: carbohydrate.
[g]DM: diabetes Mellitus.
[h]N: total number of participants.
[i]SD: standard deviation.
[h]SMS: short message service.
[i]HCP: health care provider
