# Supplementary material for: Considerations for the Development of Mobile Phone Apps to Support Diabetes Self-Management: Systematic Review
Source: JMIR Mhealth Uhealth. 2018 Jun 21;6(6):e10115. doi: 10.2196/10115 (PMC6035345; doi:10.2196/10115)
Supplement: Multimedia Appendix 2 [file mhealth_v6i6e10115_app2.pdf]

Appendix 2: Some of the developmental considerations in studies

Author, Year	Name of app / Origin (existing or developed for the study)	Users involvement	Clinical expert involvement	Functions and functionalities of the app
**Kirwan, 2013[48]**	**Glucose buddy / existing**	Not reported	Not reported	Manual logs of BG[a], diet (food items in gms), physical activities, insulin dosages and other medications into app. Analytic function which can present data in a customized graphical format. Allows users' to forward data via email. Also have a reminder function.
**Quinn, 2011[43]**	**Not specific, just called Smart Phone app / developed for the study**	Not reported	Mobile app was designed by endocrinologists and Credentialed Diabetes Educators (Quinn 2008) [54]	Manual logs of BG[a], CHO[b] intake, medications and other diabetes management information into app. In response to specific logged data, users can receive automated real-time educational, behavioural and motivational messages. Additionally, App could be used to forward data to physician.
**Charpentier, 2011 [46]**	**Diabeo / developed for the study**	Not reported	Not reported	Manual logs of BG[a], CHO[b] counts, planned activity into app to calculate insulin dose or CHO[b] adjustment. Data transfer to provider through GPRS[c] and Secured website
**Quinn, 2016 [42]**	**See Quinn 2011**	Not reported	See Quinn 2011	See Quinn 2011

**Waki, 2014[50]**	**DialBetics / not reported**	Not reported	Not reported	Automated logs of BG[a], BP[d], weight and pedometer count for physical activity level into app. Dietary evaluation of inputted meal through a feedback from a dietician (not automated; feedback takes 2 days). Analysis of BG[a] and BP[d] data with graphic outputs of measurements. Reminders for self-monitoring. Also, have a natural language processing method such that patient voice/text messages about meal and exercise that is not counted by the pedometer are sent to the server and the voice input is converted to text and matched with text in the app. Automated feedback advice on lifestyle modification in response to logged data. Alert for missed or late readings
**Orsama, 2013[49]**	**Monica / developed for the study**	Not reported	Not reported	Manual logs of BG[a], BP[d], weight and physical activity into app. Analytic function in form of graphs. Participants received automated real time education, behavioural skills and motivational messages in response to logged data.
**Kim, 2014[52]**	**Henceforth app / developed for the study**	Not reported	Not reported	Manual logs of BG[a] and BP[d] into app with automated transfer to participants hospital

**Rossi, 2010 [44]**	**Diabetes Interactive Diary / developed for the study**	Not reported	Not reported	Manual logs of BG[a] and insulin dose into app. Analysis to provide automated suggestion for insulin bolus dose and CHO[b] count. General pre-stored data on; BGa values, individualized correction factor: CHO[b] ratio set by physician, food intake and physical activity. Logged data can be sent on an average of 1-3 weeks as short messages to physician for review purpose to provide feedback to the patient via text messages. (Rossi 2009) [57]

**Holmen, 2014[51]**	**The Few Touch App / developed for the study**	12 - 15 end users were involved in the design process. Through incorporation of Focus group meetings, semi-structured interviews, usability testing, questionnaires, and paper prototyping. Results generated the design requirements and answers to research questions (Arsand, 2010)[20]	Not reported	Automated log of BG[a] readings from meter into app. Manual log of physical activity and food intake. Analytic function in form of visual graph, trend reports and feedbacks through colour coding (below normal, normal and above normal). Provided general information on disease. Also have a personal goal setting system. Automated data transfer of logged data into a secure server
**Rossi, 2013[45]**	**See Rossi , 2010**	See Rossi, 2010	See Rossi, 2010	See Rossi, 2010
**Istepanian, 2009[47]**	**Not specific just generally called m-health system / not reported**	Not reported	Not reported	Automated log of BG[a] from meter into App. Alert to remind users when measurement are due. Data transfer from mobile phone to a hospital server

[a]BG: blood glucose.
[b]CHO: carbohydrate.
[c]GPRS: General Packet Radio Service.
[d]BP: blood pressure.
