# Supplementary material for: Considerations for the Development of Mobile Phone Apps to Support Diabetes Self-Management: Systematic Review
Source: JMIR Mhealth Uhealth. 2018 Jun 21;6(6):e10115. doi: 10.2196/10115 (PMC6035345; doi:10.2196/10115)
Supplement: Multimedia Appendix 3 [file mhealth_v6i6e10115_app3.pdf]

Appendix 3: Other developmental considerations in studies and key clinical outcome-glycosylated hemoglobin.

Author, Year	Data security and privacy consideration	Pilot testing	Health behavioural theory consideration	Key Clinical Outcome ($HbA1_c$)[1]
**Kirwan, 2013[48]**	Not reported	Not reported	Not reported	The intervention group showed a significantly improved $HbA1_c$ from baseline to 9-month follow up compared to the control group.
**Quinn, 2011[43]**	Data security in the mobile phone app was not reported. But for the web-based system; BG data were captured in real time into a HIPAA[3]-compliant secured web-based system, where it is processed to receive personalized feedback. (Quinn 2008, Quinn & Gruber-Baldin 2009)[54, 92]	Pilot tested for 3 months on 30 participants with type 2 diabetes with the aim to assess the impact of the software on $HbA1_c$, and assessed provider's and patients satisfaction with the technology (Quinn, 2008)[54]	Not reported	One of the intervention group (maximal treatment) showed a significantly improved $HbA1_c$ from baseline to 12-month follow up compared to other intervention and control groups.

**Charpentier, 2011 [46]**	Data logged into app were automatically uploaded into a secured website, where they are available to the investigators at any time	Four month observational study of 35 type 1 diabetes patients. The aim was to confirm the use of personalized flexible intensive insulin therapy results in good control of the postprandial state. (Frac, 2009) [55].	Not reported	The full intervention group showed a significantly improved $HbA1_c$ from baseline to 6 month follow up compared to the control group.
**Quinn, 2016 [42]**	See Quinn 2011	See Quinn 2011	See Quinn 2011	Both the intervention and control group had reduced improvement in $HbA1_c$ after 12 months follow up.
**Waki, 2014[50]**	Not clear. Reported that participants measured data were transmitted to a server, following each new measurement that patient profile was updated which controlled access to patient's data and recorded access history.	One month pilot testing on 11 participants with type 2 diabetes. The aim was to assess the safety, usability and impact of mobile app on $HbA1_c$ and the effect on home BP[4] monitoring as a way of managing the complications related to diabetes. (Waki, 2012)[56]	Not reported	The intervention group showed a significantly improved HbA1c from baseline to 3-month follow up compared to the control group.

**Orsama, 2013[49]**	Not reported	Not reported	Information-Motivation behavioural skill model was used as the basis for the formulation of automated personalized feedback message contents	The intervention group showed a significantly improved $HbA1_c$ from baseline to 10-month follow up compared to the control group.
**Kim, 2014[52]**	Not reported	Not reported	Not reported	Both the intervention and control groups had a non- significantly improved $HbA1_c$ from baseline to 3-month follow up.
**Rossi, 2010 [44]**	Not reported	The first pilot study was done with a questionnaire to assess the feasibility and acceptability of the app. A second pilot study was done on 41 patients using DID under routine clinical practice condition were evaluated after a medium of 9 months of follow up. The aim is to investigate effectiveness of the app on metabolic control. The second pilot study was on 50 people with type 1 diabetes aged 18-65 years, with the aim of investigating the feasibility and acceptability of the app (Rossi, 2009)[57]	Not reported	Both the intervention and control groups had a non-significantly improved $HbA1_c$ from baseline to 6-month follow up.

**Holmen, 2014[51]**	Not reported	12 people with type 2 diabetes participated in the testing of the app during an average test period of 167 days (Arsand, 2009)[93]	Not reported.	Both the intervention and control groups had a non-significantly improved $HbA1_c$ from baseline to 12-month follow up.
**Rossi, 2013[45]**	See Rossi, 2010	See Rossi, 2010	See Rossi, 2010	Both the intervention and control groups had a non-significantly improved $HbA1_c$ from baseline to 6-month follow up.
**Istepanian, 2009[47]**	Not reported	Not reported	Not reported	$HbA1_c$ in the intervention and control groups remain unchanged after 9-month follow up.

[1]HA1c: glycosylated haemoglobin

[2]BG: blood glucose

[3]HIPAA

[4]BP: blood pressure
